# *catena*-Poly[[[aqua­copper(II)]-μ-hydroxido-κ^2^*O*:*O*-μ-[3-(4*H*-1,2,4-triazol-4-yl)benzoato]-κ^2^*N*^1^:*N*^2^] monohydrate]

**DOI:** 10.1107/S2414314625006388

**Published:** 2025-07-29

**Authors:** Xiao-Yu Li, Ye Zhou, Hong-Can Wang, Meng Ji, Guo-Ao Li, Ai-Xin Zhu

**Affiliations:** a Faculty of Chemistry and Chemical Engineering, Yunnan Normal University, Kunming 650050, People’s Republic of China; Vienna University of Technology, Austria

**Keywords:** crystal structure, coordination polymer, triazole-carboxyl­ate, copper complex

## Abstract

In the title compound, {[Cu(C_9_H_6_N_3_O_2_)(OH)(H_2_O)]·H_2_O]}_*n*_, the Cu^2+^ cation is situated on a twofold rotation axis and is coordinated by two triazole N atoms from two different 3-(4*H*-1,2,4-triazol-4-yl)benzoate (3-tba) ligands, by two hydroxyl O atoms and by a water O atom, forming a coordination environment inter­mediate between a square pyramid and a trigonal bipyramid. The Cu^2+^ ions are connected by 3-tba ligand and a hy­droxy group into polymeric chains parallel to [001]. O—H⋯O hydrogen bonds and C—H⋯O inter­actions consolidate the crystal structure.

## Abstract 2

The Cu^2+^ cation in the polymeric title compound has an N_2_O_3_ coordination set, inter­mediate between a square pyramid and a trigonal bipyramid.

## Graphical abstract

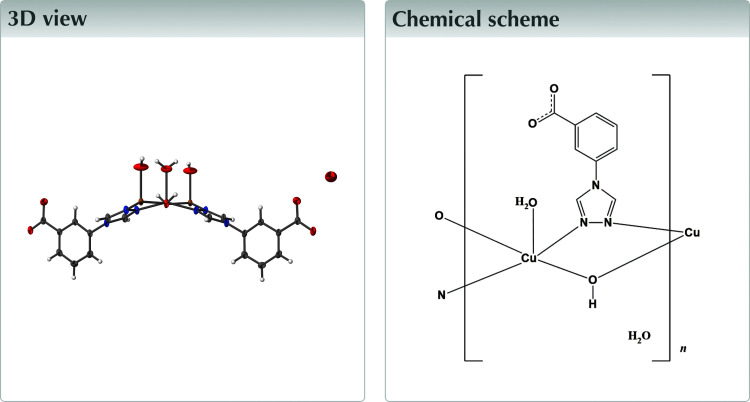

## Structure description

Coordination polymers have attracted considerable inter­est because of their distinctive topologies and various potential applications (Kitagawa *et al.*, 2004 ▸; Leong & Vittal, 2011 ▸). Since organic ligands are crucial for the assembly and structural regulation of coordination polymers, they play a decisive role in the design of such compounds. In this regard, bifunctional groups are very useful, such as triazole-carboxyl­ate ligands. For example, 4-(4*H*-1,2,4-triazol-4-yl)benzoate, 4-(1*H*-1,2,4-triazol-1-yl)benzoate, 3-(4*H*-1,2,4-triazol-4-yl)benzoate and 3-(1*H*-1,2,4-triazol-1-yl)benzoate have been used in the construction of various coordination polymers with different periodicities including dimers, chains, layers or networks (Mu *et al.*, 2014 ▸; Wang *et al.*, 2020 ▸; Yang *et al.*, 2016**a* ▸,b* ▸). In this contribution, we selected 3-(4*H*-1,2,4-triazol-4-yl)benzoate (3-tba) as a triazole-carboxyl­ate ligand, generating a new coordination polymer, {[Cu(C_9_H_6_N_3_O_2_)(OH)(H_2_O)]·H_2_O]}_*n*_, which is reported here.

**Figure 1:**
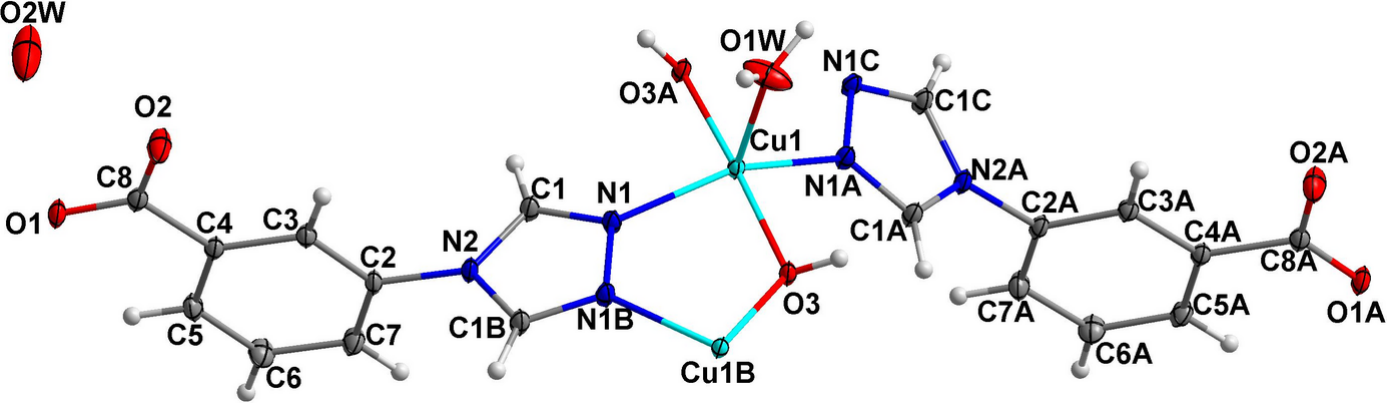
Parts of the crystal structure showing the coordination environment of Cu^2+^ in the title compound. Displacement ellipsoids are drawn at the 30% probability level. [Symmetry codes: (A) *x*, 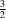 − *y*, 1 − *z*; (B) *x*, *y*, 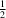 − *z*; (C) *x*, 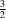 − *y*, 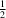 + *z*.]

**Figure 2:**
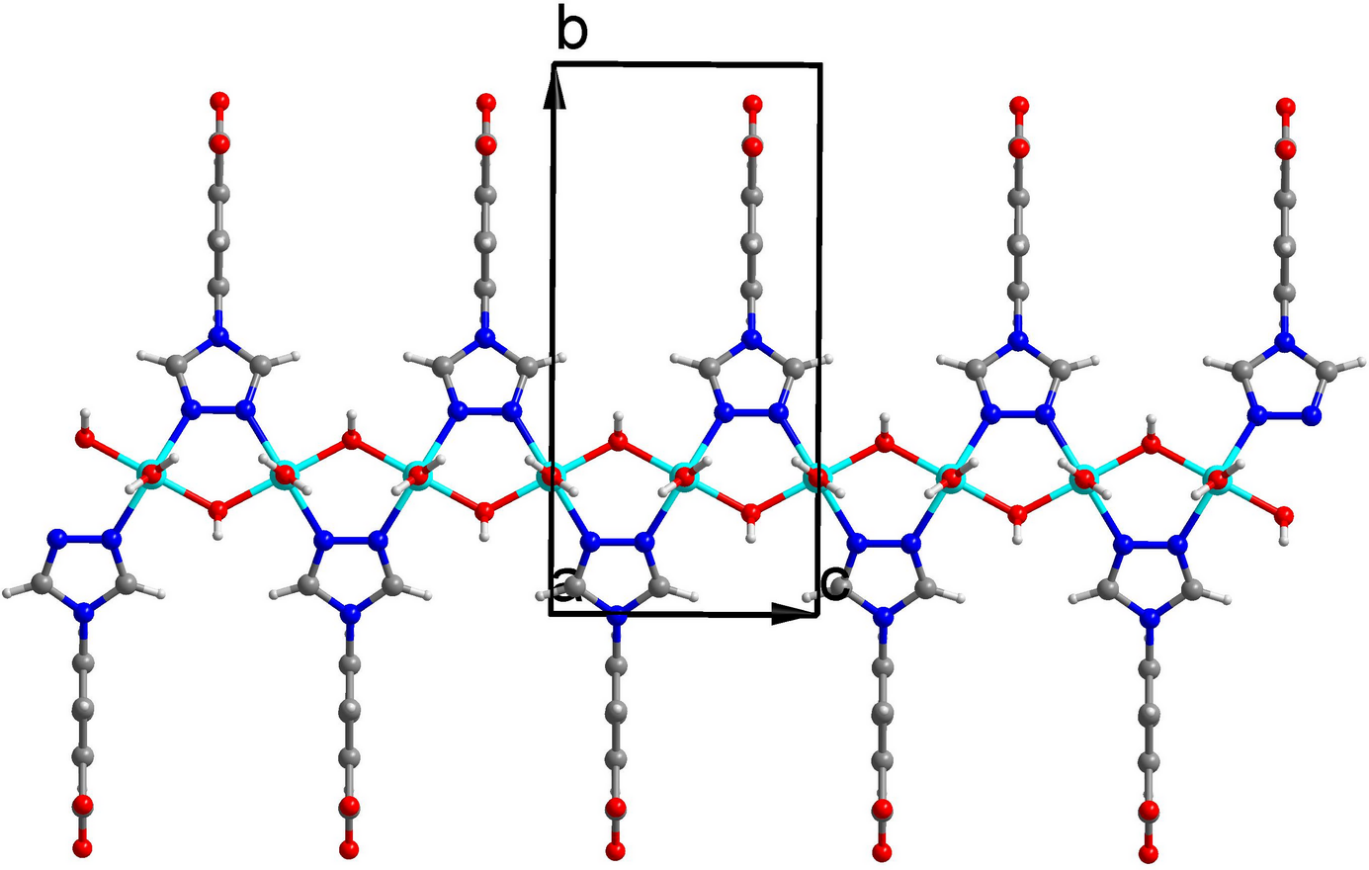
The formed polymeric chain in the title compound.

**Figure 3:**
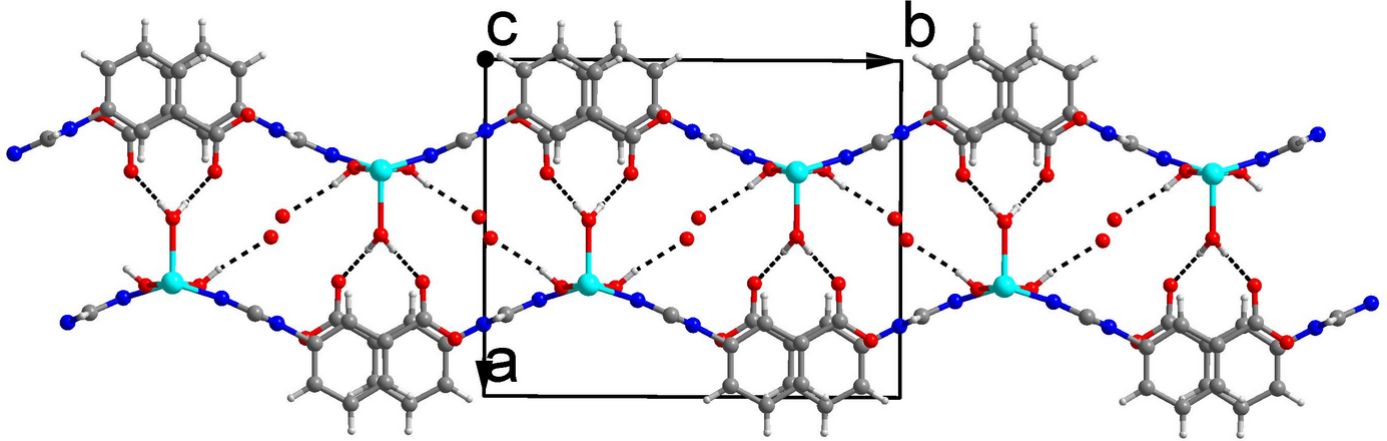
The double-sheet structure formed by O—H⋯O hydrogen-bonding inter­actions (black dashes lines) viewed along the *c* axis.

**Figure 4:**
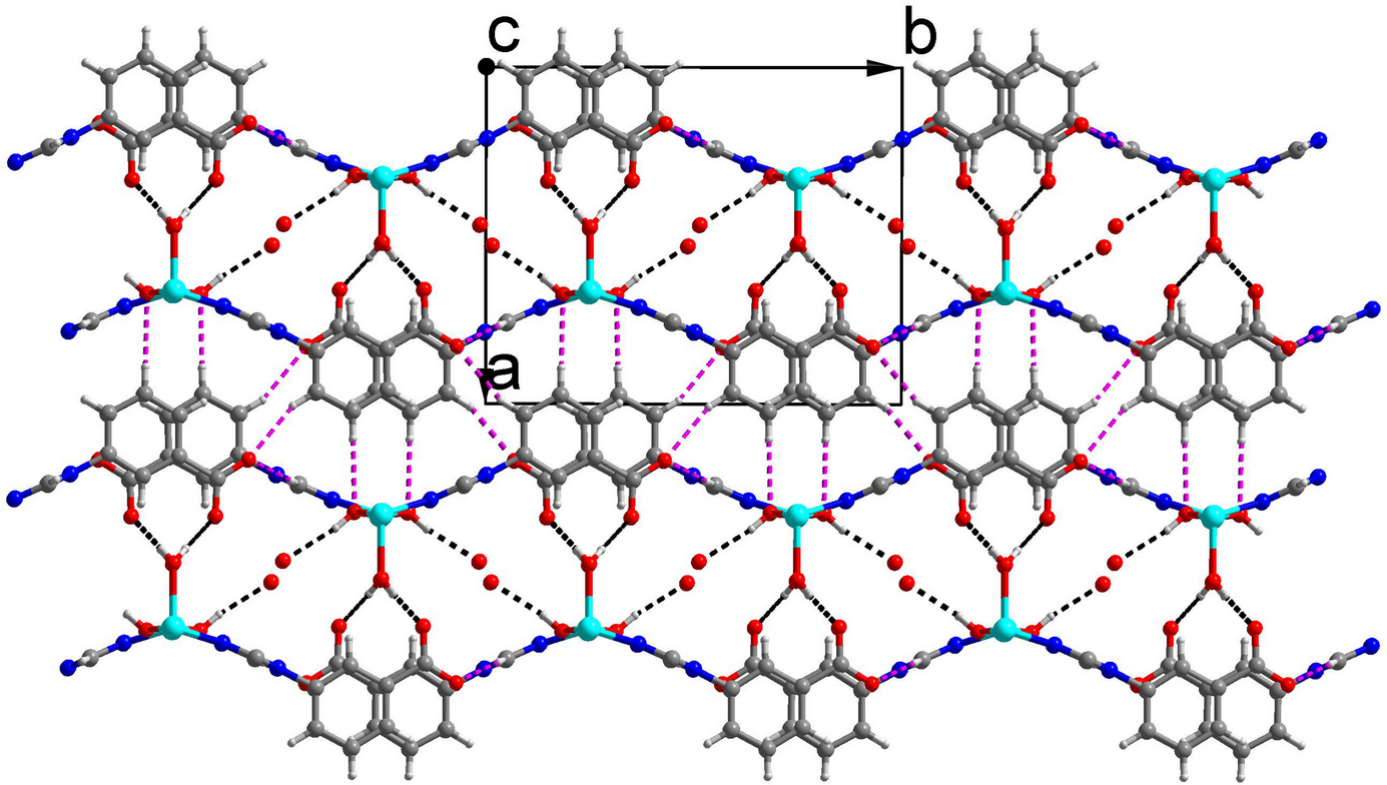
The crystal structure with O—H⋯O hydrogen bonds (black dashed lines) and C—H⋯O hydrogen bonds (purple dashed lines) viewed along the *c* axis.

All units in the crystal structure are on special positions. The Cu^2+^ ion and the coordinating water mol­ecule (O1*W)* are situated on a twofold rotation axis, whereas the hydroxyl group (O3), the non-coordinating water mol­ecule (O2*W*) and the benzoate entity of the 3-tba ligand are situated on a mirror plane, which also bis­ects the triazole entity. As shown in Fig. 1 ▸, the Cu^2+^ ion is coordinated by two nitro­gen atoms from two different 3-tba ligands and three oxygen atoms from two different hydroxyl groups and a coordinating water mol­ecule. The *τ*_5_ index (Addison *et al.*, 1984 ▸) of 0.40 indicates a coordination environment between a square pyramid (SP) and a trigonal bipyramid (TP) (extreme forms: *τ*_5_ = 0.00 for SP and 1.00 for TP). The Cu—O bond lengths are 1.9434 (9) (2× to the hydroxide O atom) and 2.186 (2) Å (to the coordinating water O atom), and the Cd—N bond length is 2.0254 (14) Å (2× to triazole N atoms). As shown in Fig. 2 ▸, the 3-tba ligand and the hydroxyl group display *μ*_2_-bridging modes to link adjacent Cu^2+^ ions into a polymeric chain extending parallel to [001]. These chain are joined *via* inter­molecular O—H⋯O hydrogen-bonding inter­actions into double sheets parallel to (100) (Table 1 ▸, Fig. 3 ▸). Since the H atoms of the non-coordinating water mol­ecule (O2*W*) were not located, the role of this mol­ecule as a donor group is unclear. However, the proximity to oxygen atoms O2 and O3 [2.746 (2) and 2.936 (4) Å] allows conclusions to be drawn as possible acceptor atoms for hydrogen bonding. The cohesion of the crystal structure into a tri-periodic framework is ensured by weak C—H⋯O inter­actions (Table 1 ▸, Fig. 4 ▸).

## Synthesis and crystallization

A mixture of Cu(NO_3_)_2_·3H_2_O (12 mg, 0.05 mmol), 3-Htba (9 mg, 0.05 mmol), water (4 ml) and ammonia solution (0.05 ml, 1 mol l^−1^) was placed in a Teflon-lined stainless steel vessel (15 ml). The vessel was sealed and heated in an oven at 393 K for 72 h, and then slowly cooled to the room temperature. Blue block-shaped crystals were harvested by filtration, washed with water and dried under ambient condition (yield 36%).

## Refinement

Crystal data, data collection and structure refinement details are summarized in Table 2 ▸. Since reliable positions of hydrogen atoms bonded to non-coordinating water mol­ecule O2*W* could not be derived from difference-Fourier maps, they were excluded from the model but are part of the formula and other structural data.

## Supplementary Material

Crystal structure: contains datablock(s) I. DOI: 10.1107/S2414314625006388/wm4229sup1.cif

Structure factors: contains datablock(s) I. DOI: 10.1107/S2414314625006388/wm4229Isup3.hkl

CCDC reference: 2473449

Additional supporting information:  crystallographic information; 3D view; checkCIF report

## Figures and Tables

**Table 1:** Hydrogen-bond geometry (Å, °)

*D*—H⋯*A*	*D*—H	H⋯*A*	*D*⋯*A*	*D*—H⋯*A*
O1*W*—H1*W*⋯O2^i^	0.85	1.90	2.746 (2)	175
O3—H3*A*⋯O2*W*^ii^	0.85	2.09	2.936 (4)	171
C1—H1⋯O1^iii^	0.93	2.19	3.027 (2)	149
C6—H6⋯O3^iv^	0.93	2.50	3.426 (3)	180
C7—H7⋯O1^v^	0.93	2.38	3.270 (3)	161

Symmetry codes: (i) 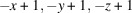; (ii) 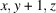; (iii) 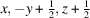; (iv) 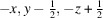; (v) 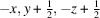.

**Table 2:** Experimental details

Crystal data	
Chemical formula	[Cu(C_9_H_6_N_3_O_2_)(OH)(H_2_O)]·H_2_O
*M*_r_	304.75
Crystal system, space group	Orthorhombic, *Pbcm*
Temperature (K)	293
*a*, *b*, *c* (Å)	11.456 (2), 14.140 (3), 6.8502 (14)
*V* (Å^3^)	1109.6 (4)
*Z*	4
Radiation type	Mo *K*α
μ (mm^−1^)	1.99
Crystal size (mm)	0.25 × 0.22 × 0.20

Data collection	
Diffractometer	Rigaku R-AXIS SPIDER
Absorption correction	Multi-scan (*ABSCOR*; Higashi, 2001 ▸)
*T*_min_, *T*_max_	0.746, 0.896
No. of measured, independent and observed [*I* > 2σ(*I*)] reflections	10406, 1374, 1209
*R*_int_	0.038
(sin θ/λ)_max_ (Å^−1^)	0.648

Refinement	
*R*[*F*^2^ > 2σ(*F*^2^)], *wR*(*F*^2^), *S*	0.027, 0.078, 1.13
No. of reflections	1374
No. of parameters	101
H-atom treatment	H-atom parameters constrained
Δρ_max_, Δρ_min_ (e Å^−3^)	0.48, −0.45

Computer programs: *RAPID-AUTO* (Rigaku, 1999 ▸), *CrystalClear* (Rigaku, 2002 ▸), *SHELXT* (Sheldrick, 2015*a* ▸), *SHELXL* (Sheldrick, 2015*b* ▸), *DIAMOND* (Brandenburg, 1999 ▸) and *publCIF* (Westrip, 2010 ▸).

## full crystallographic data

### catena-Poly[[[aquacopper(II)]-µ-hydroxido-κ2O:O-µ-[3-(4H-1,2,4-triazol-4-yl)benzoato]-κ2N1:N2] monohydrate] Crystal data

**Table:**

[Cu(C_9_H_6_N_3_O_2_)(OH)(H_2_O)]·H_2_O	*D*_x_ = 1.824 Mg m^−^^3^
*M**_r_* = 304.75	Mo *K*α radiation, λ = 0.71073 Å
Orthorhombic, *P**b**c**m*	Cell parameters from 2652 reflections
*a* = 11.456 (2) Å	θ = 5.2–54.9°
*b* = 14.140 (3) Å	µ = 1.99 mm^−^^1^
*c* = 6.8502 (14) Å	*T* = 293 K
*V* = 1109.6 (4) Å^3^	Block, blue
*Z* = 4	0.25 × 0.22 × 0.20 mm
*F*(000) = 620	

### catena-Poly[[[aquacopper(II)]-µ-hydroxido-κ2O:O-µ-[3-(4H-1,2,4-triazol-4-yl)benzoato]-κ2N1:N2] monohydrate] Data collection

**Table:**

Rigaku R-AXIS SPIDER diffractometer	1209 reflections with *I* > 2σ(*I*)
ω scans	*R*_int_ = 0.038
Absorption correction: multi-scan (ABSCOR; Higashi, 2001)	θ_max_ = 27.4°, θ_min_ = 3.4°
*T*_min_ = 0.746, *T*_max_ = 0.896	*h* = −14→14
10406 measured reflections	*k* = −17→18
1374 independent reflections	*l* = −8→8

### catena-Poly[[[aquacopper(II)]-µ-hydroxido-κ2O:O-µ-[3-(4H-1,2,4-triazol-4-yl)benzoato]-κ2N1:N2] monohydrate] Refinement

**Table:**

Refinement on *F*^2^	0 restraints
Least-squares matrix: full	Hydrogen site location: mixed
*R*[*F*^2^ > 2σ(*F*^2^)] = 0.027	H-atom parameters constrained
*wR*(*F*^2^) = 0.078	*w* = 1/[σ^2^(*F*_o_^2^) + (0.040*P*)^2^ + 0.7023*P*] where *P* = (*F*_o_^2^ + 2*F*_c_^2^)/3
*S* = 1.13	(Δ/σ)_max_ < 0.001
1374 reflections	Δρ_max_ = 0.48 e Å^−^^3^
101 parameters	Δρ_min_ = −0.45 e Å^−^^3^

### catena-Poly[[[aquacopper(II)]-µ-hydroxido-κ2O:O-µ-[3-(4H-1,2,4-triazol-4-yl)benzoato]-κ2N1:N2] monohydrate] Special details

**Table:**

Geometry. All esds (except the esd in the dihedral angle between two l.s. planes) are estimated using the full covariance matrix. The cell esds are taken into account individually in the estimation of esds in distances, angles and torsion angles; correlations between esds in cell parameters are only used when they are defined by crystal symmetry. An approximate (isotropic) treatment of cell esds is used for estimating esds involving l.s. planes.

### catena-Poly[[[aquacopper(II)]-µ-hydroxido-κ2O:O-µ-[3-(4H-1,2,4-triazol-4-yl)benzoato]-κ2N1:N2] monohydrate] Fractional atomic coordinates and isotropic or equivalent isotropic displacement parameters (Å^2^)

**Table:**

	*x*	*y*	*z*	*U*_iso_*/*U*_eq_	
Cu1	0.33189 (3)	0.750000	0.500000	0.01519 (13)	
N1	0.28663 (14)	0.63184 (10)	0.3510 (2)	0.0197 (3)	
N2	0.2127 (2)	0.49830 (15)	0.250000	0.0194 (4)	
C1	0.24192 (17)	0.55131 (12)	0.4081 (2)	0.0218 (4)	
H1	0.231667	0.533087	0.537449	0.026*	
C2	0.1535 (2)	0.40752 (18)	0.250000	0.0190 (5)	
C3	0.2189 (2)	0.32508 (17)	0.250000	0.0187 (5)	
H3	0.300014	0.327427	0.250000	0.022*	
C4	0.1603 (2)	0.23812 (17)	0.250000	0.0182 (5)	
C5	0.0387 (3)	0.23727 (18)	0.250000	0.0235 (5)	
H5	−0.000599	0.179720	0.250000	0.028*	
C6	−0.0245 (2)	0.3205 (2)	0.250000	0.0284 (6)	
H6	−0.105621	0.318531	0.250000	0.034*	
C7	0.0329 (2)	0.40733 (19)	0.250000	0.0261 (6)	
H7	−0.008844	0.463720	0.250000	0.031*	
C8	0.2265 (2)	0.14552 (17)	0.250000	0.0207 (5)	
O1	0.16904 (17)	0.07078 (13)	0.250000	0.0247 (4)	
O2	0.33717 (18)	0.14919 (15)	0.250000	0.0361 (5)	
O3	0.32341 (16)	0.81461 (12)	0.250000	0.0186 (4)	
H3A	0.371738	0.860229	0.250000	0.022*	
O1W	0.5227 (2)	0.750000	0.500000	0.0501 (8)	
H1W	0.565365	0.778680	0.582500	0.060*	
O2W	0.4674 (3)	−0.0136 (2)	0.250000	0.0820 (12)	

### catena-Poly[[[aquacopper(II)]-µ-hydroxido-κ2O:O-µ-[3-(4H-1,2,4-triazol-4-yl)benzoato]-κ2N1:N2] monohydrate] Atomic displacement parameters (Å^2^)

**Table:**

	*U*^11^	*U*^22^	*U*^33^	*U*^12^	*U*^13^	*U*^23^
Cu1	0.0241 (2)	0.00965 (18)	0.01184 (18)	0.000	0.000	−0.00046 (9)
N1	0.0339 (8)	0.0132 (7)	0.0122 (7)	−0.0031 (6)	−0.0007 (6)	−0.0003 (6)
N2	0.0297 (11)	0.0099 (9)	0.0186 (10)	−0.0024 (8)	0.000	0.000
C1	0.0359 (10)	0.0133 (8)	0.0161 (8)	−0.0029 (7)	0.0003 (7)	0.0008 (6)
C2	0.0278 (13)	0.0106 (11)	0.0186 (12)	−0.0039 (9)	0.000	0.000
C3	0.0222 (12)	0.0143 (11)	0.0196 (11)	−0.0020 (9)	0.000	0.000
C4	0.0247 (13)	0.0127 (12)	0.0172 (13)	−0.0007 (9)	0.000	0.000
C5	0.0269 (13)	0.0160 (12)	0.0277 (14)	−0.0067 (10)	0.000	0.000
C6	0.0200 (13)	0.0249 (14)	0.0403 (15)	−0.0017 (11)	0.000	0.000
C7	0.0278 (14)	0.0158 (12)	0.0348 (14)	0.0037 (10)	0.000	0.000
C8	0.0296 (14)	0.0140 (12)	0.0185 (11)	0.0005 (10)	0.000	0.000
O1	0.0344 (11)	0.0109 (9)	0.0288 (10)	−0.0020 (7)	0.000	0.000
O2	0.0267 (11)	0.0192 (10)	0.0624 (15)	0.0028 (8)	0.000	0.000
O3	0.0302 (10)	0.0106 (8)	0.0151 (8)	−0.0030 (7)	0.000	0.000
O1W	0.0236 (12)	0.091 (2)	0.0357 (13)	0.000	0.000	−0.0226 (12)
O2W	0.0488 (17)	0.0304 (15)	0.167 (4)	−0.0002 (13)	0.000	0.000

### catena-Poly[[[aquacopper(II)]-µ-hydroxido-κ2O:O-µ-[3-(4H-1,2,4-triazol-4-yl)benzoato]-κ2N1:N2] monohydrate] Geometric parameters (Å, º)

**Table:**

Cu1—O3^i^	1.9434 (9)	C3—H3	0.9300
Cu1—O3	1.9434 (9)	C4—C5	1.393 (4)
Cu1—N1^ii^	2.0254 (14)	C4—C8	1.513 (3)
Cu1—N1	2.0254 (14)	C5—C6	1.382 (4)
Cu1—O1W	2.186 (2)	C5—H5	0.9300
N1—C1	1.309 (2)	C6—C7	1.393 (4)
N1—N1^iii^	1.384 (3)	C6—H6	0.9300
N2—C1	1.359 (2)	C7—H7	0.9300
N2—C1^iii^	1.359 (2)	C8—O1	1.245 (3)
N2—C2	1.452 (3)	C8—O2	1.269 (3)
C1—H1	0.9300	O3—H3A	0.8501
C2—C7	1.382 (4)	O1W—H1W	0.8500
C2—C3	1.385 (4)	O1W—H1W^ii^	0.8499
C3—C4	1.401 (3)		
			
O3^i^—Cu1—O3	174.27 (11)	C2—C3—H3	120.7
O3^i^—Cu1—N1^ii^	86.03 (6)	C4—C3—H3	120.7
O3—Cu1—N1^ii^	92.49 (6)	C5—C4—C3	119.1 (2)
O3^i^—Cu1—N1	92.50 (6)	C5—C4—C8	119.6 (2)
O3—Cu1—N1	86.04 (6)	C3—C4—C8	121.3 (2)
N1^ii^—Cu1—N1	150.33 (9)	C6—C5—C4	121.1 (2)
O3^i^—Cu1—O1W	92.87 (5)	C6—C5—H5	119.4
O3—Cu1—O1W	92.87 (5)	C4—C5—H5	119.4
N1^ii^—Cu1—O1W	104.83 (5)	C5—C6—C7	120.2 (3)
N1—Cu1—O1W	104.83 (5)	C5—C6—H6	119.9
C1—N1—N1^iii^	107.40 (10)	C7—C6—H6	119.9
C1—N1—Cu1	131.86 (12)	C2—C7—C6	118.3 (3)
N1^iii^—N1—Cu1	120.26 (4)	C2—C7—H7	120.9
C1—N2—C1^iii^	105.7 (2)	C6—C7—H7	120.9
C1—N2—C2	127.06 (10)	O1—C8—O2	124.3 (2)
C1^iii^—N2—C2	127.06 (10)	O1—C8—C4	118.0 (2)
N1—C1—N2	109.75 (15)	O2—C8—C4	117.7 (2)
N1—C1—H1	125.1	Cu1^iii^—O3—Cu1	123.58 (9)
N2—C1—H1	125.1	Cu1^iii^—O3—H3A	108.9
C7—C2—C3	122.6 (2)	Cu1—O3—H3A	108.9
C7—C2—N2	118.0 (2)	Cu1—O1W—H1W	125.1
C3—C2—N2	119.4 (2)	Cu1—O1W—H1W^ii^	125.078 (7)
C2—C3—C4	118.7 (2)	H1W—O1W—H1W^ii^	109.8
			
N1^iii^—N1—C1—N2	0.11 (19)	C2—C3—C4—C8	180.0
Cu1—N1—C1—N2	172.00 (15)	C3—C4—C5—C6	0.0
C1^iii^—N2—C1—N1	−0.2 (3)	C8—C4—C5—C6	180.0
C2—N2—C1—N1	−175.4 (2)	C4—C5—C6—C7	0.0
C1—N2—C2—C7	87.1 (2)	C3—C2—C7—C6	0.0
C1^iii^—N2—C2—C7	−87.1 (2)	N2—C2—C7—C6	180.0
C1—N2—C2—C3	−92.9 (2)	C5—C6—C7—C2	0.0
C1^iii^—N2—C2—C3	92.9 (2)	C5—C4—C8—O1	0.0
C7—C2—C3—C4	0.0	C3—C4—C8—O1	180.0
N2—C2—C3—C4	180.0	C5—C4—C8—O2	180.0
C2—C3—C4—C5	0.0	C3—C4—C8—O2	0.0

Symmetry codes: (i) *x*, −*y*+3/2, *z*+1/2; (ii) *x*, −*y*+3/2, −*z*+1; (iii) *x*, *y*, −*z*+1/2.

### catena-Poly[[[aquacopper(II)]-µ-hydroxido-κ2O:O-µ-[3-(4H-1,2,4-triazol-4-yl)benzoato]-κ2N1:N2] monohydrate] Hydrogen-bond geometry (Å, º)

**Table:**

*D*—H···*A*	*D*—H	H···*A*	*D*···*A*	*D*—H···*A*
O1*W*—H1*W*···O2^iv^	0.85	1.90	2.746 (2)	175
O3—H3*A*···O2*W*^v^	0.85	2.09	2.936 (4)	171
C1—H1···O1^vi^	0.93	2.19	3.027 (2)	149
C6—H6···O3^vii^	0.93	2.50	3.426 (3)	180
C7—H7···O1^viii^	0.93	2.38	3.270 (3)	161

Symmetry codes: (iv) −*x*+1, −*y*+1, −*z*+1; (v) *x*, *y*+1, *z*; (vi) *x*, −*y*+1/2, *z*+1/2; (vii) −*x*, *y*−1/2, −*z*+1/2; (viii) −*x*, *y*+1/2, −*z*+1/2.

## References

[bb1] Addison, A. W., Rao, T. N., Reedijk, J., van Rijn, J. & Verschoor, G. C. (1984). *J. Chem. Soc. Dalton Trans.* pp. 1349–1356.

[bb2] Brandenburg, K. (1999). *DIAMOND*. Crystal Impact GbR, Bonn, Germany.

[bb3] Higashi, T. (2001). *ABSCOR*. Rigaku Corporation, Tokyo, Japan.

[bb4] Kitagawa, S., Kitaura, R. & Noro, S.-I. (2004). *Angew. Chem. Int. Ed.* **43**, 2334–2375. 10.1002/anie.200300610 15114565

[bb5] Leong, W. L. & Vittal, J. J. (2011). *Chem. Rev.* **111**, 688–764. 10.1021/cr100160e 20804195

[bb6] Mu, Y.-H., Ge, Z.-W. & Li, C.-P. (2014). *Inorg. Chem. Commun.* **48**, 94–98.

[bb7] Rigaku (1999). *RAPID-AUTO*. Rigaku Corporation, Tokyo, Japan.

[bb8] Rigaku (2002). *CrystalClear*. Rigaku Corporation, Tokyo, Japan.

[bb9] Sheldrick, G. M. (2015*a*). *Acta Cryst.* A**71**, 3–8.

[bb10] Sheldrick, G. M. (2015*b*). *Acta Cryst.* C**71**, 3–8.

[bb11] Wang, D., Wang, T., Zhao, P., Shi, Z. & Zhao, Q. (2020). *Inorg. Chim. Acta* **508**, 119657.

[bb12] Westrip, S. P. (2010). *J. Appl. Cryst.* **43**, 920–925.

[bb13] Yang, L.-B., Wang, H.-C., Dou, A.-N., Rong, M.-Z., Zhu, A.-X. & Yang, Z. (2016*a*). *Inorg. Chim. Acta* **446**, 103–110.

[bb14] Yang, L.-B., Wang, H.-C., Fang, X.-D., Chen, S.-J., Xu, Q.-Q., Zhu, A.-X. & Yang, Z. (2016*b*). *CrystEngComm* **18**, 130–142.

